# DeeProBot: a hybrid deep neural network model for social bot detection based on user profile data

**DOI:** 10.1007/s13278-022-00869-w

**Published:** 2022-03-12

**Authors:** Kadhim Hayawi, Sujith Mathew, Neethu Venugopal, Mohammad M. Masud, Pin-Han Ho

**Affiliations:** 1 Zayed University, Abu Dhabi, UAE; 2 United Arab Emirates University, Abu Dhabi, UAE; 3 University of Waterloo, Waterloo, Canada

**Keywords:** Social bot detection, Twitter, Deep learning, User profile metadata, LSTM, GLoVe embedding

## Abstract

Use of online social networks (OSNs) undoubtedly brings the world closer. OSNs like Twitter provide a space for expressing one’s opinions in a public platform. This great potential is misused by the creation of bot accounts, which spread fake news and manipulate opinions. Hence, distinguishing genuine human accounts from bot accounts has become a pressing issue for researchers. In this paper, we propose a framework based on deep learning to classify Twitter accounts as either ‘human’ or ‘bot.’ We use the information from user profile metadata of the Twitter account like description, follower count and tweet count. We name the framework ‘DeeProBot,’ which stands for Deep Profile-based Bot detection framework. The raw text from the description field of the Twitter account is also considered a feature for training the model by embedding the raw text using pre-trained Global Vectors (GLoVe) for word representation. Using only the user profile-based features considerably reduces the feature engineering overhead compared with that of user timeline-based features like user tweets and retweets. DeeProBot handles mixed types of features including numerical, binary, and text data, making the model hybrid. The network is designed with long short-term memory (LSTM) units and dense layers to accept and process the mixed input types. The proposed model is evaluated on a collection of publicly available labeled datasets. We have designed the model to make it generalizable across different datasets. The model is evaluated using two ways: testing on a hold-out set of the same dataset; and training with one dataset and testing with a different dataset. With these experiments, the proposed model achieved AUC as high as 0.97 with a selected set of features.

## Introduction

The rise of technology, mobile smart devices and high-speed internet have increased the use of OSNs. As per the study by Kemp (2021), there are 4.2 billion social media users around the world, which is more than 53 percent of the world’s total population. Facebook, which is the biggest social media network worldwide, has 2.8 billion monthly active users, and Twitter has 330 million monthly active users (Tankovska 2021). Twitter provides an open platform to express opinions publicly that gives Twitter an extra potential to influence the public. The influence of OSNs on the public encouraged the emergence of machine accounts or social bots.

**Fig. 1:**
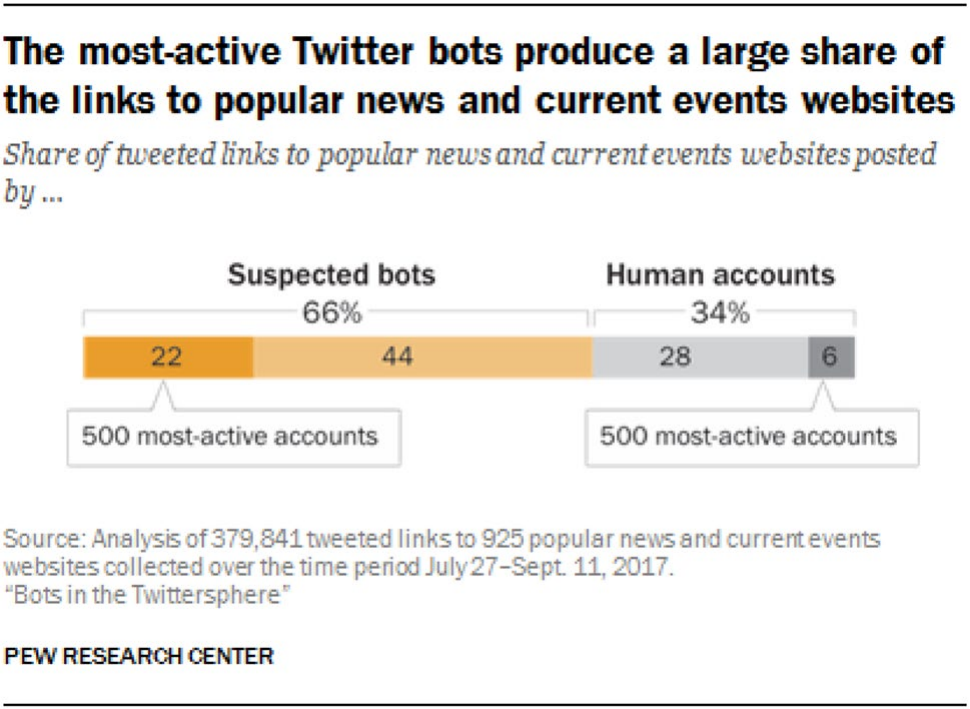
Proportion of tweeted links to popular websites by bots and human (Stefan et al. 2018)

As per Ferrara et al. (2016), a social bot is a computer algorithm that automatically produces content and interacts with humans on social media, trying to emulate and possibly alter their behavior. As per Varol et al. (2017), up to 15% of Twitter users are social bots. Recently, a study by Carnegie Mellon University researchers has shown that among 200 million tweets discussing coronavirus, 82% of the influential re-tweeters were bots (Virginia 2020). Also, in the study by Pew Research Center, it has been estimated that two thirds of tweeted links to popular websites are posted by automated accounts and not by humans. As per their study, bots shared 66% of all tweeted links to popular websites (Stefan et al. 2018). The statistic in this study is given in Fig. 1. Social bots have a positive impact when they act as helpers in aggregating and delivering news feeds, or as automatic responders for customer care. But they have also been misused to spread rumors and fake news to mislead the public. For example, in Mumbai, rumors were spread in social media that the vaccines were a plot by the government to sterilize Muslim children that led to only 50% of those who were expected to be vaccinated to actually get the vaccine (Larson 2020). Work by Chang et al. (2021) studied the effect of social bots and social media manipulation around two major events of 2020, namely the 2020 US Presidential Election and Covid-19 pandemic. It has been found that bots generated much higher volumes of election-related tweets per capita and also tweet supporting a specific political line. Also, social bots are found responsible for posting and amplifying less credible information regarding Covid-19 pandemic (Yang et al. 2020a). Social bots are also a major source of climate change disinformation that might drain support from policies to address rising temperatures (Corbin 2021).

There are several types of bots posing different behaviors. For example, traditional spambots generate a lot of content-promoting products. Social spambots tend to attack or support political candidates, and fake followers tend to have aggressive following patterns (Sayyadiharikandeh et al. 2020). The different behavioral patterns of bots make it difficult to detect, if we look into it through the same lens. Most of the works for detecting bots concentrate on specific behavioral patterns that make it difficult to detect all types of bots. For real-time bot detection, a generalizable bot detection method is required.

In this work, we are proposing a novel framework to detect bots in Twitter. The deep neural network-based framework, DeeProBot, is designed to make it generalizable to detect bots across unseen datasets. Only the user profile information from the Twitter account is used for detecting bots. These are the features that we get from the user object of Twitter Application Programming Interface (Twitter API). These features include the username, screen name, tweet count, followers count, friends count, listed count, user created date, description, location, url, verified flag, etc. Detecting bots using just the profile information instead of going deep into the content created by the user reduces the overhead of extra feature extraction and processing. As we discussed, bots evolve over time. A traditional bot reveals much less personal information through the profile. They have random names for the profile with missing details. At the same time, recently evolved sophisticated bots have their profile similar to legitimate human-operated accounts. In such a scenario, we put forward a novel idea of using the text in the Twitter account profile description as a feature, which is usually avoided in relevant works. The description text always contains useful information in detecting whether an account is a bot or a human. This led to the design of a deep neural network-based architecture where the description text is embedded using GloVe (Pennington et al. 2014) pre-trained word embedding weights and processed using LSTM units. Other features like followers count and following count are equally important because bots show the pattern of following large numbers of accounts while having a smaller number of followers. To capture all these features, we developed a hybrid architecture to process both numerical and text features. The major contributions of this work are as follows:

- We have designed and developed DeeProBot, a generalizable deep neural network-based framework that uses profile metadata information for detecting bots in Twitter achieving better performance compared to state-of-the-art methods.
- The proposed framework utilizes the potential of the profile description text as a feature and GloVe word embedding in detecting bots.
- We analyze the effect of feature selection in the model performance.
- We have proposed a hybrid design of deep neural network model using LSTM and dense layers to handle mixed input types.
- We have performed cross-domain performance evaluation of DeeProBot by training the model on a set of heterogeneous Twitter datasets and testing the performance of the model on four other heterogeneous datasets not used in training.

The rest of the paper is organized as follows. We discuss the research works related to bot detection in Sect. 2. Section 3 provides details of the datasets used. Section 4 describes the DeeProBot framework, including the feature engineering module, model architecture and algorithms explaining the framework. The experiments and evaluations are given in Sect. 5. Finally, Sect. 6 concludes the work and discusses the future research directions.

## Related work

The bot detection techniques in OSNs can be broadly classified as (a) Graph-based methods (b) Crowdsourcing methods and (c) Machine Learning methods (Alothali et al. 2018). The graph-based methods capture the network communication patterns of the users to distinguish them as genuine or bot (Dorri et al. 2018; Abu-El-Rub and Mueen 2019). However, graph-based methods mostly depend on assumptions. The computational cost can also be high based on the size of the network. In crowdsourcing methods, human effort and expertise are utilized in annotating user accounts as genuine or bot (Wang et al. 2012, 2014). This method is time-consuming and is prone to human error as it involves human intelligence. In the literature, researchers have mostly used machine learning methods for bot detection. Machine learning methods involve learning from data. Here, features are extracted from Twitter user accounts, which represent the behavioral patterns of the users. These features are fed to the network to classify them as bot or human. In this work, we are using a machine learning-based model to detect bots.

A user’s profile, content and temporal features can be extracted from Twitter (Zahra et al. 2020; Shukla et al. 2021). In Sayyadiharikandeh et al. (2020), the diversities of different types of bots are handled by training classifiers specialized for each class of bots, and a bot-score is calculated for each classifier. The classifier that outputs the highest bot-score determines the corresponding class. They have also done a cross-domain analysis of their classifier by testing it on separate datasets to demonstrate the generalizability of the model. They have used a high-dimensional feature set consisting of 1200 features from six categories: metadata from accounts and friends, retweet/mention networks, temporal features, content information, as well as sentiment. Considering a rich feature set that includes an account’s actions and social connections improves accuracy but reduces scalability (Yang et al. 2020b). In the work by Yang et al. (2020b), only the profile information of the user account is considered for training a Random Forest classifier. They proposed a scalable and generalizable bot detection method and used a data selection criterion to find the best model. Most of the methods that use only the metadata information from the user profile are trained using Random Forest or Adaboost classifiers (Daouadi et al. 2020; Kondeti et al. 2021). Deep learning techniques are not much explored when using these sets of features.

Deep learning techniques for bot detection usually use content information like tweet text along with temporal data or a combination of all types of features from Twitter account. In the work by Wu et al. (2021), the detection of social bots from Sina Weibo, one of the most popular Chinese OSNs in the world, uses 30 features from four categories, namely metadata-based, interaction-based, content-based, and timing-based. These are then fed to a deep neural network (DNN) model consisting of a residual network (ResNet), a bidirectional gated recurrent unit (BiGRU), and an attention mechanism. They obtained an accuracy of 0.98. Since it has been developed for Sino Weibo, the performance of the same for Twitter data needs to be checked. The work by Braker et al. (2020) uses a multi-layer perceptron (MLP) network to detect bots in Twitter which is trained on a lower-dimensional feature set extracted from account metadata and tweet metadata. They obtained an accuracy of 0.92 and a lower recall percentage leaving scope for improvement. The work presented in Kudugunta and Ferrara (2018) used a contextual-LSTM network to learn the tweet text along with the metadata features to detect bots. They have not considered the description feature along with the metadata features and also have not tested the cross-domain performance of the model on separate datasets. Similarly, there are several deep learning works that use the tweet text for detecting bots in Twitter (Dukić et al. 2020; Mou and Lee 2020). In this work, we are not focusing on tweet text. In a study by Cresci (2020), unsupervised approaches have been found effective in detecting groups of coordinated bots. But such methods are slow as there is a need to consider a group of accounts for detecting coordinated activity. Currently, this is not under our scope of work as we are detecting bots based on individual account features.

To the best of our knowledge, there is no deep learning approach that considers only the profile information for bot detection. The proposed work uses data extracted exclusively from the profile metadata of Twitter account. We use the profile information in a comprehensive way that includes numerical, categorical and text features from the user profile. Additionally, better insights on the model performance could be provided, if researchers analyze the performance of their model on datasets that are different from the training dataset in terms of data crawling or annotation strategies (Sayyadiharikandeh et al. 2020; Yang et al. 2020b). Rauchfleisch and Kaiser (2020) has studied that bot detection technique like botometer gives imprecise results on bot datasets with bot behavior different from the ones it is trained for. So, a cross-domain analysis gives a better understanding on the generalizability of the detection framework which is not presented for other deep learning works doing bot detection.

## Datasets

We used the datasets provided by the public bot repository of the botometer.^1^ Bot repository is a centralized place to share annotated datasets of Twitter social bots. The list of the datasets that we used, with a brief description, is given in Table 1. The training set is formed by merging the datasets specified under training set. A combination of heterogenous datasets as the training data helps in including bots evolved at different time periods and with different behavioral patterns. Using such a training set makes the model generalizable to detect different types of bots. This combination is selected based on the work by (Yang et al. 2020b). They have employed a data selection technique to find the best subset of data that creates a model, which performs better in terms of accuracy and generalizability. *midterm-18*, *cresci-rtbust* and *gilani-17* are hold-out datasets considered separately for cross-domain testing. Another test dataset is formed by combining *botwiki* and a subset of *verified* to make it a balanced dataset of bots and humans. Here, each dataset is labeled using different strategies and methods. Also, they are collected at different time frames. These factors make the datasets characteristically different from each other as studied in (Yang et al. 2020b).

**Table 1:** List of datasets and its description

Dataset	Description	#Bots	#Human
Training set			
varol-icwsm (Varol et al. 2017)	Manually labeled accounts sampled from different Botometer score deciles	674	1471
cresci_17 (Cresci et al. 2017a, 2017b)	This dataset provides four classes of accounts namely genuine users, social spambots, traditional spambots and fake followers	10,894	3474
Celebrity (Yang et al. 2019)	Data based on accounts selected among celebrities	0	5917
botometer-feedback (Yang et al. 2019)	Data obtained by manually labeling accounts flagged by feedback from Botometer users	139	379
political-bots (Yang et al. 2019)	Data based on politics-oriented bots shared by a Twitter user	61	0
Test set			
Botwiki (Yang et al. 2020b)	Data is based on the botwiki.org archive of self-identified bot accounts	697	0
Verified (Yang et al. 2020b)	Data obtained by collecting verified accounts from the streaming API	0	1986
midterm-18 (Yang et al. 2020b)	Data based on political tweets collected during 2018 US midterm elections	42,445	8092
cresci-rtbust (Mazza et al. 2019)	A manually annotated dataset based on Italian retweets between June 17–30, 2018	353	339
gilani-17 (Gilani et al. 2017)	Data based on accounts collected using twitter streaming API that were grouped and manually annotated	1089	1413

### Dataset separability

**Fig. 2:**
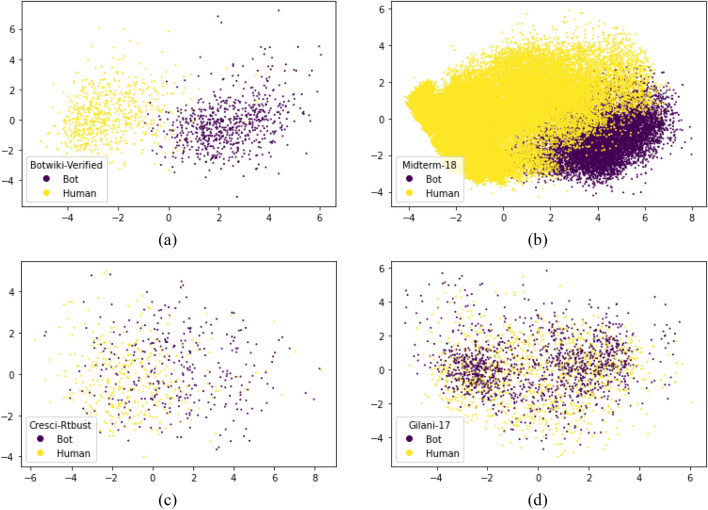
PCA plots for test datasets. This plot shows the separability between each class in the dataset with given set of features. The plots in **a** and **b** show easily separable datasets, while that in **c** and **d** show more complex datasets in terms of separability

In this section, we analyze the separability of the test datasets with respect to a given set of features to demonstrate the difficulty in classifying the dataset into different classes (here the classes are ‘bot’ and ‘human’). We applied Principal Component Analysis (PCA) on the numerical features of the four test datasets. PCA is used for dimensionality reduction, where a higher-dimensional feature set is reduced to a two-dimensional feature set. This can be plotted in a 2D plane. As a preprocessing step, power fit transform is applied to the numerical features before applying PCA to deal with the skewness in the data (Yeo and Johnson 2000). Figure 2 shows the plot obtained after applying PCA. The separability decreases from (a)–(d).

The plot for *botwiki-verified* dataset shows high separability between bots and humans with the selected set of features, whereas for *cresci-rtbust* and *gilani-17,* the points are overlapping indicating a difficult classification task.

These two datasets consider diverse types of accounts that are manually annotated based on different types of behavior. For example, *gilani-17* dataset is formed by grouping Twitter accounts into four groups based on the number of followers and then manually annotated based on some account properties and rules (Gilani et al. 2017). For *cresci-rtbust*, data are collected from 10 M retweets, and their retweet pattern is analyzed. Human accounts are labeled based on clusters with normal retweet patterns, and those with suspicious retweet patterns are labeled as bot. (Mazza et al. 2019). Yang et al. (2020b) has presented a more detailed analysis of the performance of these datasets. This analysis is supported by the empirical results reported in Sect. 5.3.5, which indicates that less separability leads to a lesser prediction accuracy.

## Proposed work

**Fig. 3:**
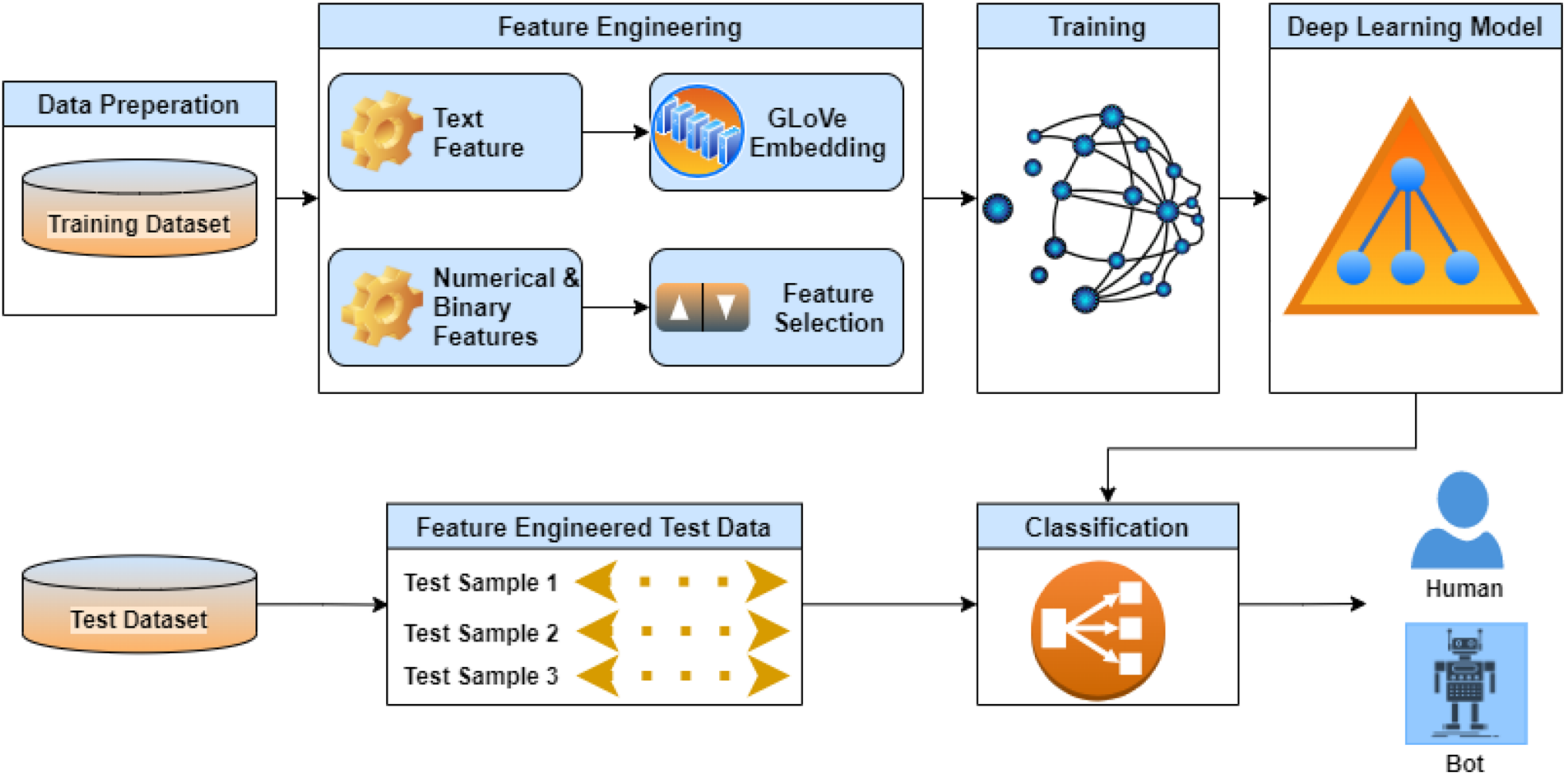
Architecture of the DeeProBot framework

Figure 3 shows the overall architecture of the DeeProBot framework, which consists of several modules, including data preparation, feature engineering and training the DNN model. Data preparation consists of building the training dataset and test datasets. This is already discussed in the previous section. Feature engineering consists of preprocessing and preparing the features. A separate preprocessing technique is applied based on the type of the feature. The preprocessed text feature undergoes the GloVe embedding. Also, a feature selection method is applied on the numerical and binary features. This reduces the dimension of the feature vector by selecting the best subset of features. These processed data are then used to train a deep neural network model to classify bots and humans. Each of these modules is explained in the following subsections.

### Feature engineering

The features are extracted from the user profile of Twitter account, which are given in Table 2. These features are the metadata from the user account. When considering the extraction of data from Twitter using Twitter API, the profile metadata of the user is contained in the user object of Twitter API, and the timeline information of the user that includes user’s recent tweets and mentions are contained in the user tweet timeline object. Instead of considering different types of features like content-based and interaction-based features that are extracted from the user timeline, we only took the user profile features primarily to investigate how only profile information can contribute to efficient bot detection and thereby avoiding the extra overhead in extracting data from Twitter. Moreover, for all our datasets except *varol-icwsm*, the user profile features were available to download directly from the bot repository. We used the Twitter API to extract the account features of *varol-icwsm* database. Unlike other works that use user account metadata features, we consider the description text of user profile as a feature in the proposed DeeProBot framework. We also derived several features from the metadata features. The derivations are inspired from the works by Inuwa-Dutse et al. (2018) and Yang et al. (2020b). The explanation of some of the derived features is given here, and the others can be easily understood from the description in the table.

**Table 2:** List of features used and its description

Feature name	Feature type	Feature description
Raw features		
Statuses_count	Numerical	The number of Tweets (including retweets) issued by the user
Followers_count	Numerical	The number of followers this account has
Friends_count	Numerical	The number of users this account is following
Favourites_count	Numerical	Number of favorites obtained from metadata
Listed_count	Numerical	The number of public lists that this user is a member of
Default_profile	Binary	When True, indicates that the user has not altered the theme or background of their user profile
Verified	Binary	When True, indicates that the user has a verified account
Description	Text	The user-defined text describing their account
Derived Features		
User_age	Numerical	The age of the account in days obtained by taking the difference of data collection date and account created date
Tweet_freq	Numerical	Statuses_count/user_age
Followers_growth_rate	Numerical	Followers_count/user_age
Friends_growth_rate	Numerical	Friends_count/user_age
Favourites_growth_rate	Numerical	Favourites_count/user_age
Listed_growth_rate	Numerical	Listed_count/user_age
Followers_friends_ratio	Numerical	Followers_count/friends_count
Screen_name_length	Numerical	Length of screen name
Name_length	Numerical	Length of name
Description_length	Numerical	Length of description
Num_digits_in_screen_name	Numerical	Number of digits in screen name
Num_digits_in_name	Numerical	Number of digits in name
Screen_name_freq	Numerical	Mean bigram frequency of characters in screen name
Screen_name_entropy	Numerical	Entropy of screen_name
Name_entropy	Numerical	Entropy of name
Description_entropy	Numerical	Entropy of description string
Name_sim	Numerical	Similarity between screen name and name
Names_ratio	Numerical	Ratio of length of screen_name to length of name

The derived feature, *screen_name_freq,* is computed from the *screen_name* of the account based on its bigram character combination.

$${\text{screen}}\_{\text{name}}\_{\text{freq}} = \frac{{\mathop \sum \nolimits_{i = 1}^{K} C\left( {b_{{\text{i}}} } \right)}}{K}$$

where $b_{i}$ is the $i^{th}$ bigram in the *screen_name.* $C\left( {b_{i} } \right)$ is the total count of the specified bigram in the *screen_name,* and $K$ is the total number of unique character bigrams in the *screen_name*.

*screen_name_entropy*, *name_entropy* and *description_entropy* are obtained by calculating the entropy of the specific character sequence. Entropy of a sequence $x$ is given by,

$$E\left( x \right) = \frac{H\left( x \right)}{{\left| x \right|}}$$

where $\left| x \right|$ is the length of the sequence, and $H\left( x \right)$ is the Shannon entropy of the sequence given by,

$$H\left( x \right) = - \mathop \sum \limits_{i = 1}^{K} p\left( i \right)*\log_{2} p\left( i \right)$$

where $p\left( i \right)$ is the probability of $i^{th}$ unique character in sequence $x$, and $K$ is the total number of unique characters in sequence $x$.

The *name_sim* feature represents the similarity between the *screen_name* and *name* given in the account profile. This is a numerical value given by,

$${\text{name}}\_{\text{sim}} = \frac{2M}{N}$$

where $M$ is the number of matches and $N$ is the total number of elements in both sequences. Table 3 shows an example of the *screen_name* and *name* of both human and bot classes and their corresponding derived features from these values.

**Table 3:** Derived features from *name* and *screen_name*

	Human	Bot
*Screen_name*	ShaneRWatson33	Tennessee_hire
*Name*	Shane Watson	Tennessee jobs
*Screen_name_freq*	1	1
*Screen_name_entropy*	0.241	0.192
*Name_entropy*	0.257	0.197
*Name_sim*	0.692	0.571

#### Numerical and binary feature preparation

This section discusses the preprocessing done for numerical and binary features, and the feature selection method applied on this set of features.

##### Numerical feature processing

Numerical feature processing involves missing value imputation and standardization. We encountered missing values only with the *description_length* feature as the description field is null for some accounts. In such cases, the *description_length* is given a value of zero. Further, the numerical features like *followers_count* are skewed. The *followers_count* feature has a minimum value of 0 and a maximum value of 108,990,846. To deal with the skewness, standardization is applied to all the numerical features (Shukla et al. 2021). We obtain the standard score, $z$ for an input $x$ by,

$$z = \frac{{\left( {x - u} \right)}}{s}$$

where $u$ and $s$ are mean and standard deviation of the sample, respectively.

##### Binary feature processing

Binary features have discrete data that can take only two different values. The features like *verified* and *default_profile* take the values True or False, and hence they are binary. These feature values are encoded so that the encoded value $z$ for input $x$ is given by,

$$z = \left\{ {\begin{array}{*{20}l} 1 \hfill & {{\text{if}}} \hfill & {x = {\text{True,}}} \hfill \\ 0 \hfill & {{\text{if}}} \hfill & {x = {\text{False}}} \hfill \\ \end{array} } \right.$$

The same principle is applied to the *Label* field where, the class ‘*bot’* is encoded as 1 and ‘*human’* is encoded as 0.

##### Feature selection

Feature selection allows to select the best subset of features that improves the model’s performance. This helps in reducing the complexity of the model by reducing the dimension of the input vector (Khalid et al. 2014). Further, scalability is a common concern when using deep learning architectures. We assume that the training of the model would be done offline, and therefore, scalability will not be much issue if the model is only used for testing. In such a scenario, feature selection also helps in improving the scalability of model in real-time data classification. There are filter methods and wrapper methods for feature selection. Filter methods try to find the relevance of features based on statistical tests on the features. This method is independent of its performance on a machine learning algorithm (Hall 1999; Dash and Liu 2003). Wrapper methods select the best subset of features based on its performance on a machine learning algorithm (Kohavi and John 1997). It has been seen that wrapper methods improve the performance of the model (Xue et al. 2015). We use backward elimination for feature selection which is a wrapper-based method.

Feature selection by backward elimination is a sequential feature selection method (Ferri et al. 1994). In the backward elimination method, a particular machine learning model is trained and cross-validated iteratively. It starts with considering all the features and at each iteration, the least significant feature is eliminated so that the cross-validation performance of the model is improved. This is repeated until it reaches an optimum number of features as required. We used a Random Forest model with threefold-cross-validation starting with all 24 features except the description feature to find the best ten features among those. We used the Random Forest model instead of our own model to reduce the risk of overfitting and to reduce the time complexity associated with the sequential feature selection approach. The final set of features after feature selection is given in Table 4.

**Table 4:** Final set of features after feature selection

Final set of features	Feature type
Statuses_count	Numerical
Followers_count	Numerical
Friends_count	Numerical
Favourites_count	Numerical
Listed_count	Numerical
Tweet_freq	Numerical
Num_digits_in_name	Numerical
Screen_name_freq	Numerical
Name_entropy	Numerical
Description_entropy	Numerical
Description	Text

#### Text feature preparation

In the DeeProBot framework, we consider the description given in the user profile as a feature to the DNN model. The description of the account gives valid information on the behavior of the account holder. Table 5 shows the example of description text for both classes. A more detailed analysis of the description field is given in Sect. 5.3.2. The description feature is a text field. Here, each word in the description is encoded as an integer before feeding to the embedding layer. This is also known as tokenizing the text. The feature engineering pipeline for the description field involves:

- *Missing value imputation*: The description field of the Twitter account is nullable, and because of that the data will have null values for this feature. The null values are replaced with a default string, *‘missing.’* The default string is meant to communicate that the data is missing. However, the description*_length* feature for null description values remains zero.
- *Cleaning the text*: Cleaning the text involves removing URLs, hashtags, mentions, and also emojis, emoticons and special characters in the description text (Srijith 2020).
- *Removing stop word*: Stop words are those words in the vocabulary which are common in usage but carry less information. These words are removed so that the vocabulary size is reduced, and importance is given to words containing high-level information (Srijith S 2020).
- *Converting to lowercase:* All the text is converted to lowercase and extra spaces are removed.
- *Tokenize the text*: Tokenizing the text involves encoding each word in the description text by a unique integer based on the vocabulary index of each word. A vocabulary index is created by assigning an integer value to each unique word in the vocabulary. This value is given based on the word’s frequency of occurrence. So that, the most frequent word gets an index value of 1 and so on. This can be performed using the Tokenizer API provided with Keras (Chollet 2016). So, after tokenizing, a sample text is converted to the following form:
  $$sample bot decription \to \left[ {25 30 265} \right]$$
- *Pad/truncate to equate the length*: The model expects equal length input to the embedding layer. So, each tokenized sequence is padded with 0 to make all the sequences to be same in length.

**Table 5:** Example of description text for bot and human classes

Class	Description text
Bot	I am a member of a network of stock investing educators. Check out more stock tips & resources on investing-information.com
	The only website dedicated to streaming The Inbetweeners for FREE!!!!!!!
Human	The official Twitter of fashion designer Vivienne Tam
	Lawyer, dog lover, passionate about music, politics, literature, cricket, and art in that order

##### Glove: Global vectors for word representation

We use an embedding layer on top of the LSTM layers to convert the words in the text to real numbered vectors. This helps in representing the text in such a way that similar words have a similar representation based on its semantic meaning. The weights of this layer can be randomly initialized and updated during model training. If the training data is small, the model may not be able to learn the embeddings to capture its semantic relationships.

A more efficient performance can be obtained if we use pre-trained weight vectors that are built on a larger training set. GLoVe pre-trained word embeddings are made available by the authors of Pennington et al. (2014), in their website.^2^ GloVe is an unsupervised learning algorithm for obtaining vector representations for words. The GloVe is trained on the nonzero entries of a global word–word co-occurrence matrix, which tabulates how frequently words co-occur with one another in a corpus. GloVe is a log-bilinear model with a weighted least-squares objective. The training objective of GloVe is to learn word vectors such that their dot product equals the logarithm of the word’s probability of co-occurrence. They provide embeddings specific for Twitter data. The pre-trained GLoVe model for Twitter is trained on 2 billion tweets with 27 billion tokens. Four models with different word vector dimensions are available. We used the 50-dimensional model after experimenting with each model, and the results are analyzed in Sect. 5.3.2. Hence, each word will be represented by a vector of length 50.

### Model architecture

**Fig. 4:**
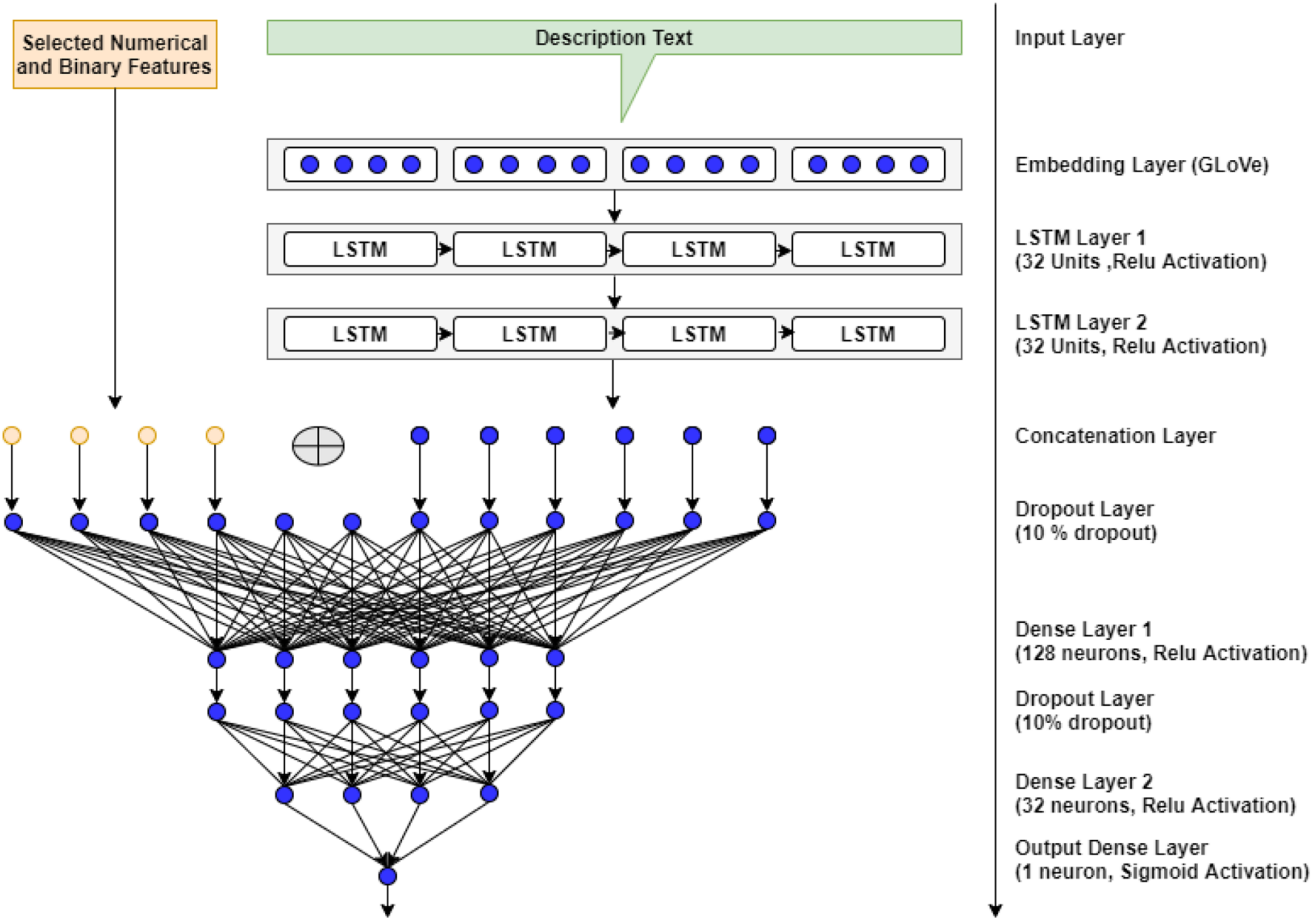
Deep learning model architecture for bot detection

Our proposed framework detects bots using the profile features including the description text. We have designed a deep neural network-based architecture as shown in Fig. 4. A DNN-based model has been proven to perform better in NLP-based tasks. At the same time, we need to learn from the non-text features like followers count and friends count, which are considered as the potential features for bot detection. Keeping that in mind, we designed a hybrid model with two input layers. One is for the description text and the other for the set of numerical features obtained after feature selection. The tokenized description text is represented by a vector of 30 integers. This dimension is selected based on the word count in the description text. In our dataset, the longest description consists of 49 words. But a word count above 40 is found only in two cases. So, we have chosen 30 as the integer vector size because 95% of the description has 30 or fewer words. This ensures that we include the needed information in the description text without causing over-clipping of data. This sequence is then converted to GLoVe embedded word vectors using the embedding layer. After embedding, each word in the sequence is represented by a vector of 50 real numbers. This embedded description text is then processed by two long short-term memory (LSTM) layers with 32 units each. LSTM is a specific type of recurrent neural network (RNN) architecture, which is mainly used in processing time-series data (Hochreiter and Schmidhuber 1997). It has been successfully used in natural language processing (NLP) where text data is processed to solve different types of tasks like classification and prediction (Wang et al. 2018; Yilmaz and Toklu 2020). The output from the LSTM units is concatenated with the ten-dimensional numerical features obtained after feature selection. This concatenated input vector is fed through two dense layers having 128 and 32 neurons respectively, with ‘relu’ activation. A dense layer is a fully connected layer that generally performs a linear operation on its inputs and is passed through a nonlinear activation function. Then, it is fed to the final dense layer with one neuron with sigmoid activation function. This layer classifies the input as a human or bot. Our architecture combines the strength of LSTM units to learn from the description text and that of the dense layers to learn from other features.

#### Strategies for improving generalizability

In many cases, the bot detection frameworks that perform exceptionally on the validation data fail to perform so in an unseen dataset or with a separate bot distribution. This is because of the lack of generalizability of the model. Deep learning models are prone to overfitting which reduces the generalizability of the model. In the proposed DeeProBot framework, the model is designed to reduce overfitting so that it can perform well on the hold-out datasets. We have incorporated dropout layers and regularization techniques to address the problem of overfitting and, thereby achieving a high degree of generalization.

- *Dropout*: Adding dropout is a simple and effective solution for reducing overfitting and improving generalization. Dropout means dropping out units in a neural network. Randomly chosen units based on a dropout rate are temporarily removed from the network during training (Srivastava et al. 2014). Adding a dropout layer has the effect of combining different neural network architectures efficiently like that of an ensemble model. In this design, we have introduced dropout layers with a 10 percent dropout rate before each of the first two dense layers. The parameter value is set based on the performance of the model.
- *Regularization*: One of the disadvantages of LSTM is that they are prone to overfitting. This can be controlled by introducing regularization to the LSTM layers. Regularization adds an extra penalty term to the error function and this term controls the coefficients from taking extreme values. Hence, regularization is a technique that discourages learning of a complex model and in turn avoids the risk of overfitting. We used activity regularization in the LSTM layer so that the weight and bias values are adjusted to keep the output small. There are several types of regularizers that we can use, and the commonly used ones are L1 regularizer and L2 regularizer (Hoerl and Kennard 1970; Tibshirani 1996). L1 regularizer adds absolute value of the magnitude of layer output as a penalty term to the loss function, whereas L2 regularizer adds squared magnitude of layer output as penalty term to the loss function. We use the combination of both L1 and L2 regularizers in the LSTM layer as the activity regularizer (Zou and Hastie 2005). The regularized loss will be defined as,
  $${\text{Loss}}_{{{\text{reg}}}} = {\text{Loss}} + l1\sum \left| x \right| + l2\sum x^{2} ,$$
  where $\left| x \right|$ is the absolute value of magnitude of layer output and $x^{2}$ is the squared magnitude of layer output. $l1$ and $l2$ are the regularization factors.
- *Early Stopping*: As the training of the network proceeds, there is a point of time when the network starts deviating from the goal by the noise in the training dataset. Thereby, it reduces the generalizability of the network and starts overfitting. More precisely, from this point, as the network is learning increasingly from training data, the training loss continues to decrease, but the validation loss starts increasing. This results in poor model performance across unseen datasets. As a solution to this, we can tune the hyperparameter, *number_of_epochs,* to select the parameter value that gives the best model. A more efficient and simpler alternative is to introduce an early stopping strategy (Prechelt 1998; Yao et al. 2007). In this method, at each epoch of training, we validate the performance of the model on a hold-out validation set and track the validation metric. The training is stopped as soon as the validation metric starts deteriorating. A more stable stopping criterion is to monitor the performance for some more additional epochs to confirm that the performance is not getting better. We have used the early stopping strategy to decide when to stop the model training. At each epoch, the validation loss is monitored. The validation loss is expected to decrease on each epoch. Once the validation loss stops improving after a certain number of epochs, it can be a sign of overfitting. The validation loss is monitored over some more epochs characterized by the *patience* parameter to check if it improves further. If the validation loss still does not improve, the training process is halted.

### DeeProBot algoithms

In this section, we summarize the whole training phase of the DeeProBot framework with the help of algorithms. The testing and analysis will be discussed in the following sections.

Algorithm 1 describes the text feature processing submodule. The function in each step is also described in detail in Sect. 4.1.2. Here, the input is the text data from the training set. In step 1, we define a for loop to handle each user’s text description separately. If the text description is empty, it is replaced by a default string D in step 3, as explained in Sect. 4.1.2. In step 5, the text sequence is filtered to remove all hashtags, mentions, symbols, emoticons, stop words and extra white spaces. The filtered text is then converted to lowercase in step 6. Now, we have the updated text data containing filtered text sequences. Step 8 creates an object of the Keras tokenizer class, which is fit on the filtered text data in step 9. Step 10 tokenizes the text using the tokenizer object where each word in the text is integer encoded. Finally in step 11, the tokenized text is padded to a finite length, L so that all sequences have equal length. This returns the processed text as output.
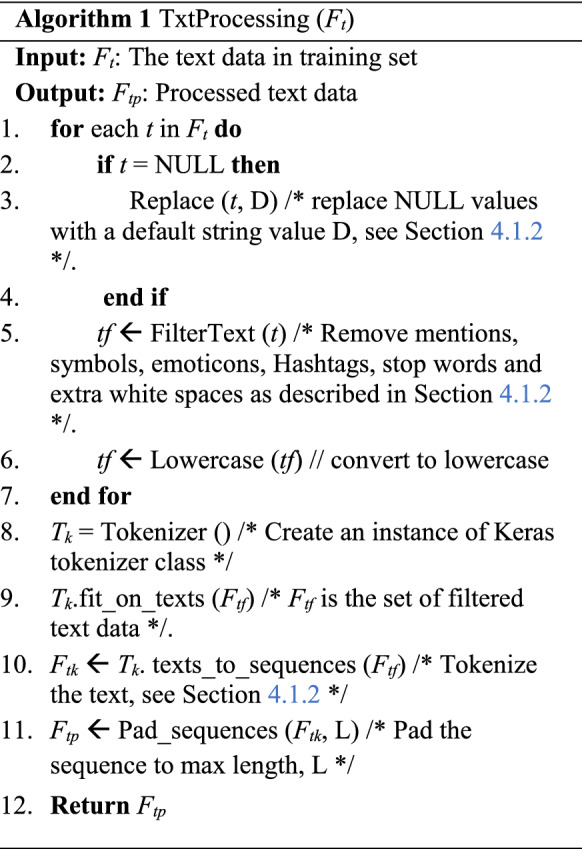


We describe the steps in creating the deep neural network model in Algorithm 2. Here the inputs, *D1* and *D2* are the dimensions of text feature and non-text feature, respectively. Steps 1 and 2 define the input layers for text and non-text features based on their respective dimensions. In step 3, embedding layer is defined to embed the text feature using GloVe. Step 4 presents the LSTM layers to process GloVe embedded data. The concatenation layer in step 5 is to concatenate the non-text input, *I*_*x*_ with the LSTM output, *L*_*l*_. Step 6 defines the dropout and dense layers to process the concatenated output. The output layer in step 7 is a dense layer with sigmoid activation function. Step 8 creates the model, and this is returned as the output. The detailed architecture of the model is given in Fig. 4 and explained in Sect. 4.2.
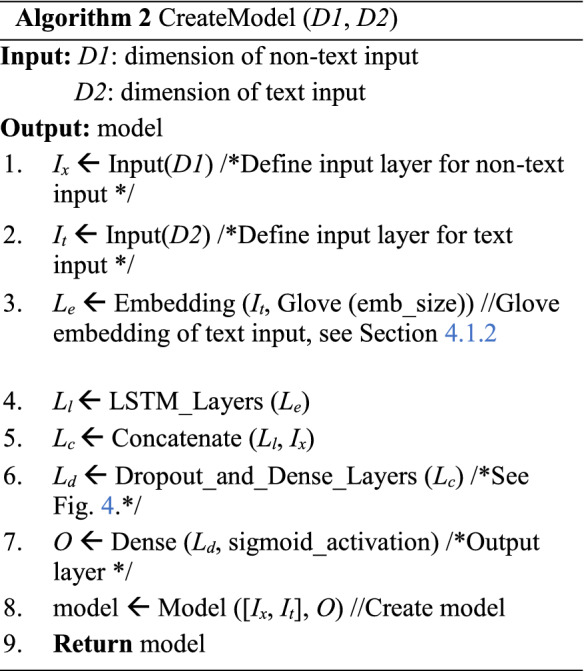


Algorithm 3 describes the whole DeeProBot framework. The input is the training data that consists of numerical features, binary features and text features along with the labels. In step 1, the numerical data is standardized, and in step 2, the binary data is encoded as described in Sect. 4.1.1. In step 3, we get the processed text data following the steps in Algorithm 1. In step 4, we use the processed numerical and binary data to get the K selected subset of features based on feature selection explained in Sect. 4.1.1. In steps 5 and 6, we select the subset of numerical and binary data based on the selected set of features. Steps 7 and 8 update the training set based on the selected features and the processed text data. Step 9 creates the model as described in Algorithm 2. Steps 10 and 11 compile the model and train the model and return the trained model as output.

## Experiments

This section describes the experimental setup, implementation details and result analysis of the work done. The DeeProBot framework focuses on detecting bots in Twitter.

### Hardware and software

We have implemented the whole framework on Intel Core i7 CPU with 8 GB RAM under Windows 10 with 64-bit Operating System. We used the Anaconda platform for developing the work. Anaconda is an open-source platform for developing data science and machine learning projects using Python or R. It also supports different Python libraries like NumPy, Pandas, Scikit-Learn, Keras etc. and various Integrated Development Environments (IDEs) like ‘spyder’ ‘jupyter notebook’ etc. for code development. We have implemented the application using Python 3.6. The deep learning module is implemented using Keras Library with TensorFlow as backend.
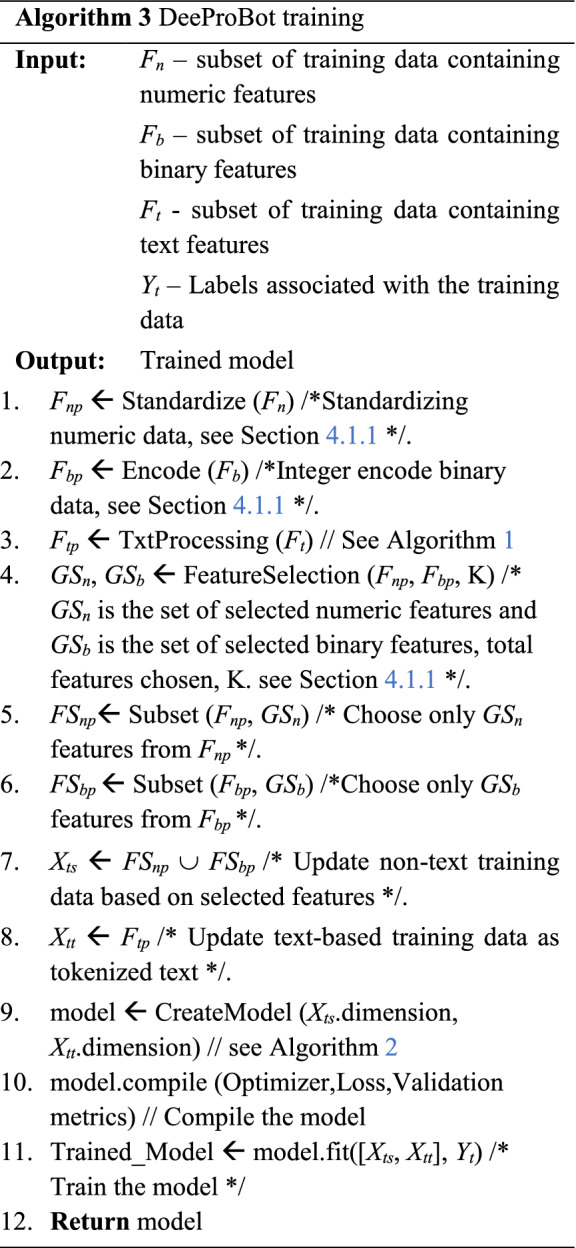


### Parameter setting

**Fig. 5:**
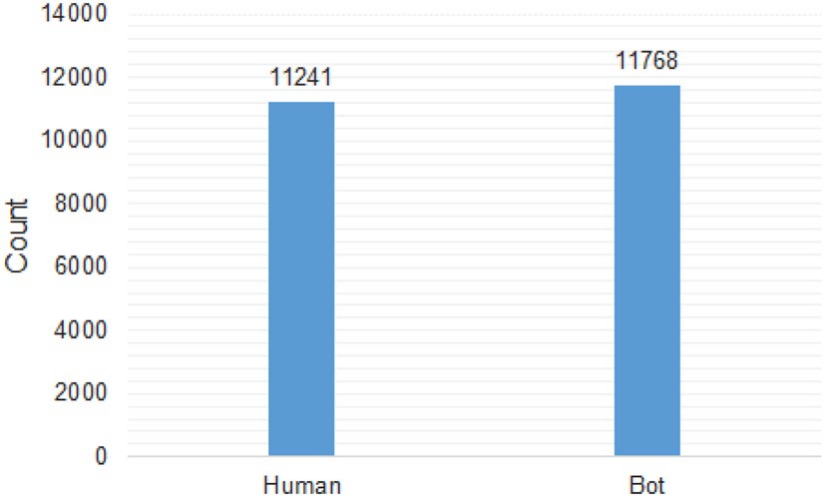
Bar plot showing the number of samples in each class of training dataset. This shows a balanced distribution of bots and human in training set

The DeeProBot framework is a bot detection framework based on DNN where the network learns from the profile metadata information of Twitter account. Our training set is obtained by merging five different datasets. The merged training dataset has a balanced set of bots and humans as shown in Fig. 5. The DNN model is trained using the description feature and the ten numerical features obtained by backward elimination feature selection, as explained in Sect. 4.1.1. Other hyperparameters used in the model are given in Table 6.

**Table 6:** Hyperparameters for neural network model

Hyperparameters	Value
Batch size	40
Learning rate	0.001
Optimizer	Adam
Loss	Binary cross entropy
L1_L2 Regularizer	L1 = 0.01, L2 = 0.001
Dropout rate	0.1
Early stopping metric	Validation loss
Early stopping patience	5

### Evaluation

Throughout this section, we report the results obtained by different evaluation strategies, such as how feature selection has helped in the better generalization of the model, the effect of using description text as a feature to detect bots etc. We also observe the impact of using the GLoVe word embedding, to represent the description feature. Finally, we compare our results with the baseline techniques in terms of feature richness, hold-out test set performance, and cross-domain performance.

We use the Area Under Curve (AUC) of Receiver Operator Characteristic (ROC) as a metric for evaluating the performance of the model. ROC is a probability curve that plots the True Positive Rate (TPR) against False Positive Rate (FPR) at various threshold values.

$${\text{TPR}} = \frac{{{\text{TP}}}}{{{\text{TP}} + {\text{FN}}}}$$

$${\text{FPR}} = \frac{{{\text{FP}}}}{{{\text{FP}} + {\text{TN}}}}$$

where ${\text{TP}}$, ${\text{FP}}$ and ${\text{FN}}$ stand for True Positive, False Positive and False Negative counts, respectively. We have done analysis on a hold-out test set, and we have done cross-domain performance analysis. Twenty percent of data from the training set is kept aside as a hold-out test set, and performance analysis is done on that data. For cross-domain analysis, we test the performance of the model on four test datasets that are not used for training or validation.

#### Effect of feature selection

The current results are obtained using a reduced set of 11 features from profile metadata that includes ten numerical features and the description text. Table 7 shows a comparison of the performance of the model with and without feature selection. Even though the AUC on the hold-out test set has been reduced after the feature selection, the performance on unseen datasets has been mostly improved. The performance on the *midterm-18* dataset has been improved by 10% and that of *gilani-17* has been improved by 5%, while the hold-out test score and the performance of *botwiki-verified* have been slightly decreased (only by 1- 2%). The raw set of features were contributing to the slightly higher performance of the model on *botwiki-verified* dataset. However, it has been shown that, by backward elimination feature selection, we can reduce the dimensionality of the feature set and significantly improve the performance of the model on majority of the datasets.

**Table 7:** Result analysis based on feature selection

	*Hold-out set* (AUC)	*Botwiki-verified* (AUC)	*Midterm-18* (AUC)	*Cresci-rtbust* (AUC)	*Gilani-17* (AUC)
Without feature selection	0.98	0.99	0.86	0.69	0.62
With feature selection	0.97	0.97	0.96	0.72	0.67

#### Effect of adding description as a feature

As per our knowledge, the previous works for bot detection simply excluded the description text while considering some of its statistical measures like length and entropy as features. The description field usually has the content that depicts the behavior of the account owner. Using the description text for bot detection is not much analyzed in the literature. We have done a study on the description field to check how it can contribute to the bot detection framework.

**Fig. 6:**
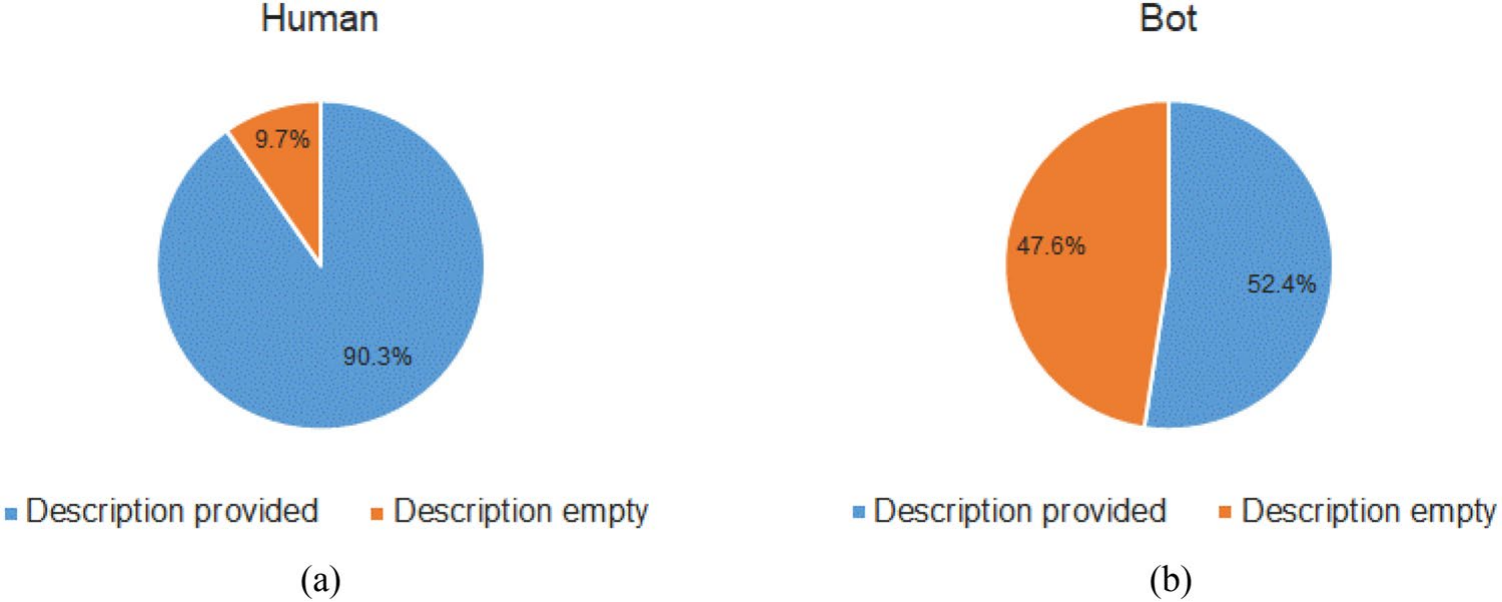
Proportion of empty description field in both human and bot classes. **a** Shows the description field status of human class where only 9.7% of users have an empty description field. **b** Shows the status of bot class where almost half of the users have an empty description text

**Fig. 7:**
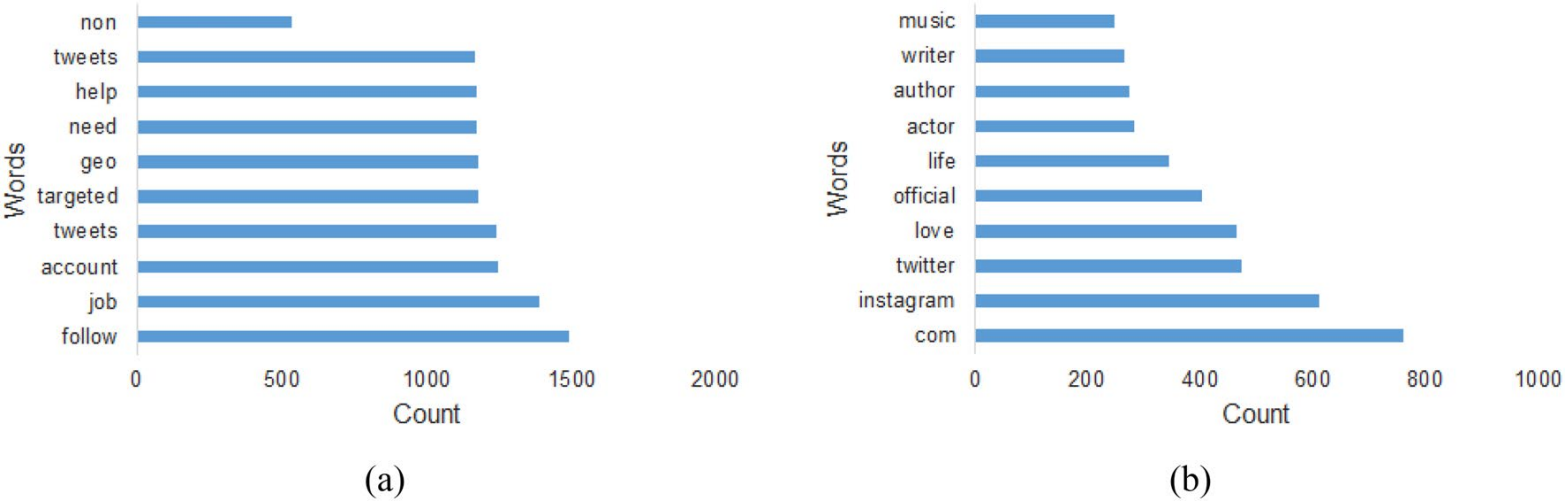
**a** Shows the top 10 common words in Bot Class. **b** Shows the top 10 common words in Human Class. The words and their frequencies are entirely different for both classes

In Twitter, the user provides his/her bio in the description field during account creation. This field can be left blank too. Figure 6 shows the proportion of empty description text in both human and bot classes. Only 10 percentage of human class has an empty description field, whereas the field is empty for approximately 50 percentage of bots. We also analyzed the most frequent words appearing in the description text for both human and bot classes. To do the analysis, we removed the stop words from the description text that appears frequently but provide less information. Figure 7 shows the top 10 common words along with their frequency counts found in both human and bot classes. It can be seen that the top 10 common words and their frequencies are significantly different for both classes. This clearly states that the inherent behavior in description text can definitely contribute to distinguish between human and bot classes.

We utilized the potential of LSTM cells to process the description text. This method has been adopted in processing tweet texts in other bot detection works. We also analyze how adding the description as a feature and its embedding strategy affects the results of DeeProBot. To get the model performance without description feature, we only used the dense layers in the model to process the numerical features. Table 8 shows it clearly that the performance of the model on unseen datasets, consistently improved after adding the description feature. Even though it does not show any improvement on the hold-out test set, it can improve the generalization capability of the model. After adding the description text as a feature, we analyzed the performance of the model without GLoVe embedding. In the model without GLoVe embedding, the embedding layer is initialized with random weight values. It will learn an embedding for all the integer encoded words in the training set when model training is done. The results presented in Table 8 show the effect of using GLoVe pre-trained word embedding weights instead of training the embedding weights from randomly initialized values. We used the GLoVe model for Twitter data that was trained on 27 billion unique tokens extracted from a set of 2 billion tweets. It can be seen from the results that training our own embedding layer has degraded the performance as the network failed to learn the embedding from our limited set of vocabulary. Also, we tested with all the four GLoVe models provided and the best performance was obtained by using the 50-dimensional model.

**Table 8:** Result analysis of adding description text as a feature

	*Hold-out set* (AUC)	*Botwiki-verified* (AUC)	*Midterm-18* (AUC)	*Cresci-rtbust* (AUC)	*Gilani-17* (AUC)
Without description	0.97	0.95	0.94	0.66	0.63
Without GLoVe embedding	0.93	0.78	0.84	0.68	0.60
With 25D GLoVe embedding	0.96	0.95	0.93	0.69	0.60
With 50D GLoVe embedding (DeeProBot)	0.97	0.97	0.96	0.72	0.67
With 100D GLoVe embedding	0.97	0.97	0.94	0.71	0.64
With 200D GLoVe embedding	0.96	0.96	0.94	0.66	0.67

#### Comparison with other baselines

Here, we compare the performance of our model with the work by Yang et al. (2020b) and other machine learning techniques. In the work by Yang et al. (2020b), they proposed a data selection method to find the best subset of data for training, to get the best generalizable model. The results are shown in Table 9. They have used Random Forest for the classification task and trained the model with different combinations of training datasets. They found the best combination of training dataset based on cross-validation and cross-domain performance. We adopted their best-performing training dataset combination for our work too. We reproduced their results by using the same dataset and the same set of features with Random Forest classifier. The AUC values obtained are different from those mentioned in the base paper. This can be due to the changes in the updated database files from the repository. We also extracted the data of *varol-icwsm* through Twitter API. This data may also have undergone changes from that used in their work. Based on the results we got, we can say that the feature selection technique along with data selection can improve the generalization ability of the model. Even though the performance of the DeeProBot model is slightly lower (only 1% reduction) on the hold-out set and lower by 2% in the performance of *gilani-17* dataset, the proposed model achieves significantly higher AUC in *cresci-rtbust* dataset (by 24% improvement) and 3% higher AUC in *botwiki-verified* dataset. We also tested our data with other machine learning techniques like Adaboost, Gated Recurring Unit (GRU) and Convolutional Neural Networks (CNN). Even though Adaboost is showing a better performance with the hold-out test set, its performance drastically decreases for cross-domain test datasets. GRU and CNN networks are also showing poor performance compared to the proposed framework. Based on these comparisons, it is evident that the DeeProBot framework shows an overall better cross-domain performance.

**Table 9:** Comparison of DeeProBot framework with other methods based on AUC

Method	Feature set	*Hold-out set* (AUC)	*Botwiki-verified (*AUC)	*Midterm-18* (AUC)	*Cresci-rtbust* (AUC)	*Gilani-17* (AUC)
Yang KC et al.(Yang et al. 2020b)	20 features based on user profile	0.98	0.94	0.96	0.48	0.69
AdaBoost	11 features based on user profile	0.99	0.82	0.95	0.42	0.59
GRU	11 features based on user profile	0.96	0.90	0.84	0.61	0.61
CNN	11 features based on user profile	0.95	0.82	0.83	0.63	0.66
DeeProBot	11 features based on user profile	0.97	0.97	0.96	0.72	0.67

#### Effect of overfitting reduction mechanisms

**Fig. 8:**
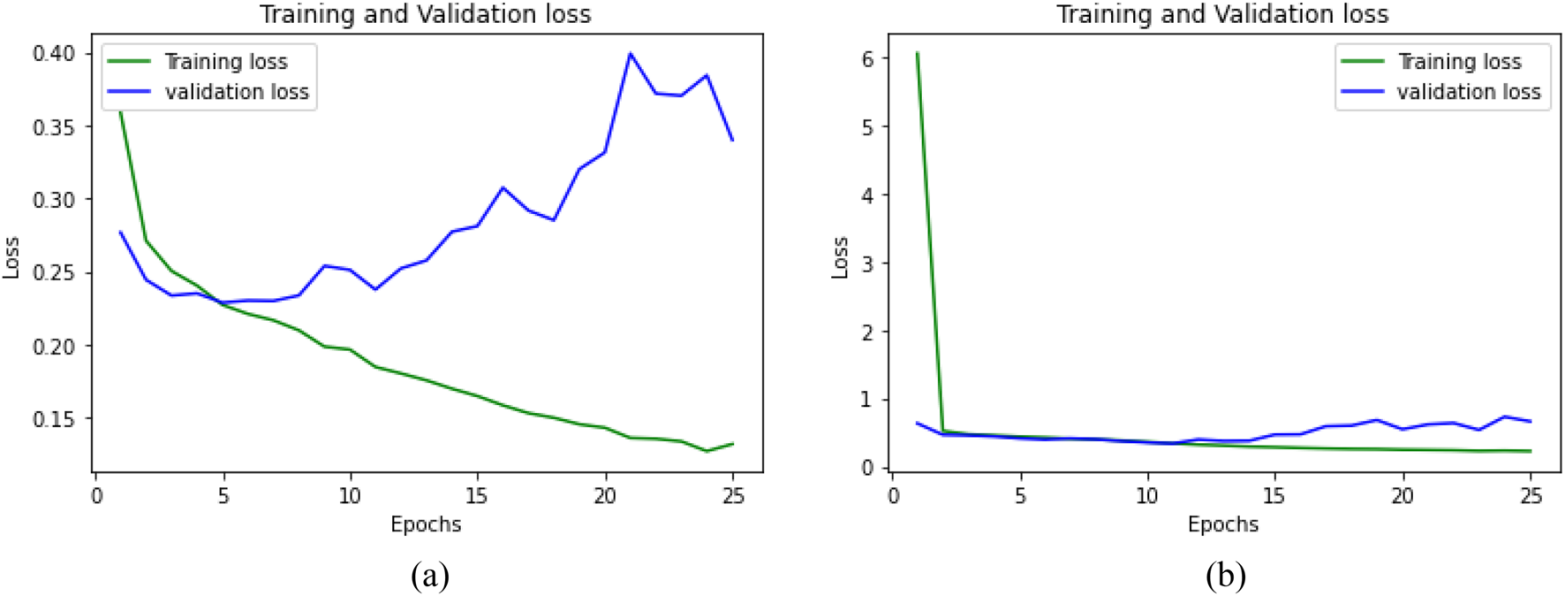
Training loss versus validation loss based on training history for 25 epochs. **a** Learning curve for the model without dropout and activity regularizer. The model fails to fit without these regularization strategies **b** Learning curve for model with dropout and activity regularizer shows the model fits its best through initial epochs

Figure 8 shows the performance of the proposed model based on the training history of the model. Here, we plot the training loss versus validation loss for 25 epochs. The plot on the left shows the performance of the model without dropout and activity regularizer. It can be seen that the model fails to fit properly without these strategies. Even though the training loss decreases per epoch, the validation loss tends to increase. This means that the model is overfitting with the training data and hence reduces the generalizability of the model. The plot in the right shows the performance of the model with dropout and activity regularizer. Here, we can see that the overfitting problem has been controlled by introducing dropout and regularization. The model sustains a good fit performance until a certain number of epochs. Even though the model tends to overfit after almost 15 epochs, the training process will be accordingly halted by the early stopping strategy. This shows that introducing regularizers, dropout and early stopping helped us in getting the best generalizable model.

#### ROC curve analysis on test data

**Fig. 9:**
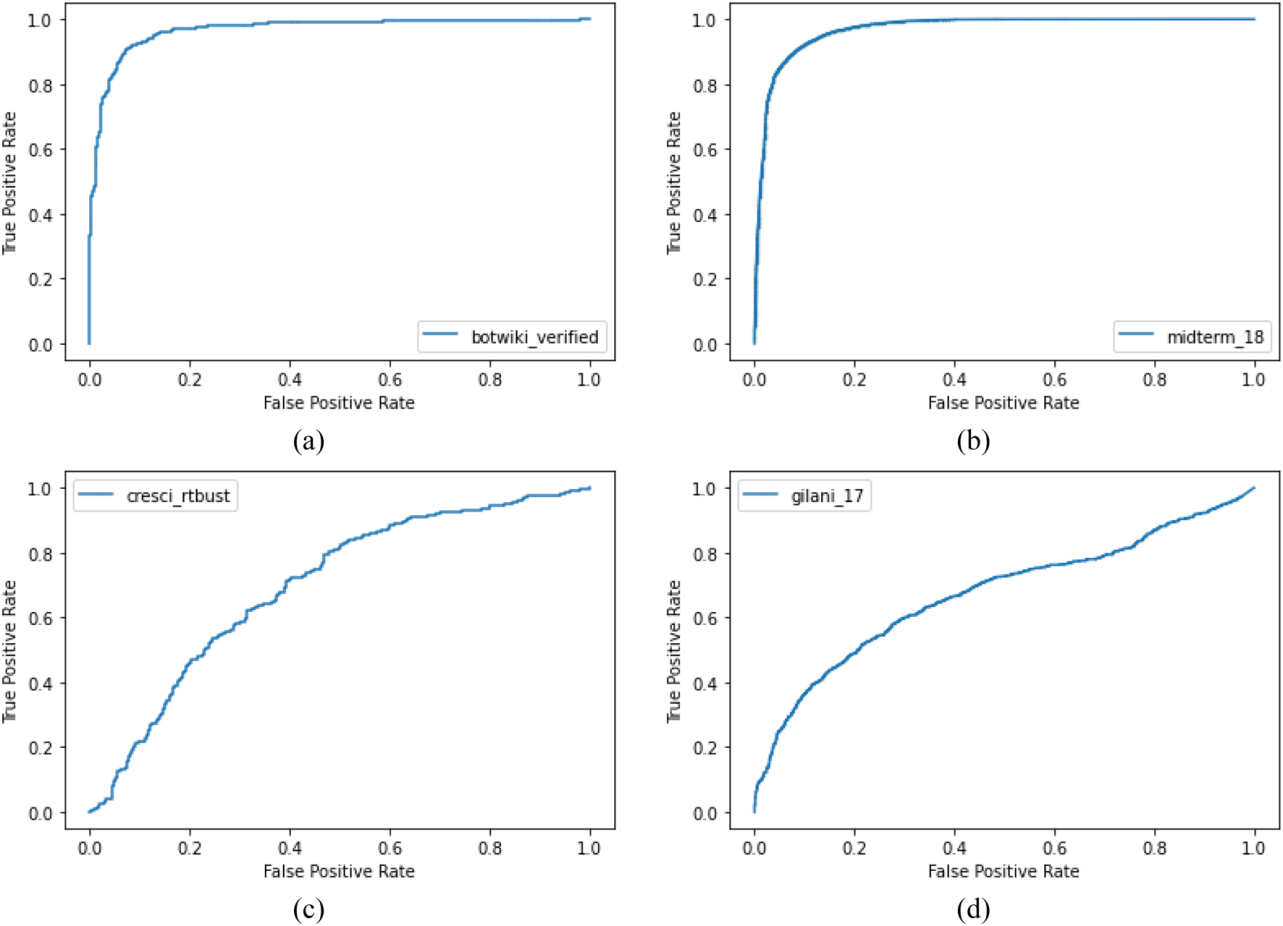
ROC curves for the performance of DeeProBot on test datasets. **a** ROC of *botwiki-verified* and **b** ROC of *midterm-18* show superior performance while **c** ROC of *cresci-rtbust* and **d** ROC of *gilani-17* which are separably complicated datasets show a lower performance

Figure 9 shows the ROC-AUC curves for the test datasets. Considering the overall performance across the four different test datasets, the model has performed well with *botwiki-verified* and *midterm-18* datasets. The performance is low for *cresci-rtbust* and *gilani-17* datasets. In the dataset analysis explained in Sect. 3.1, these two datasets were found to be the most complicated datasets in terms of separability. *cresci-rtbust* dataset is annotated based on group activity and retweet behavior. Our work tries to find individual bot accounts and these types of bots may not appear suspicious when considered individually. This led to the lower AUC values for *cresci-rtbust*. *gilani-17* dataset includes sophisticated bots from different categories annotated by human. One of the reasons for a lower AUC for *gilani-17* is the absence of such types of bots in the training dataset. Secondly, we assume that the selected features for our work may not be sufficient to distinguish bot accounts from this dataset and may require some other high-level features to learn the peculiar bot behavior. Third, *gilani-17* dataset is annotated manually. Annotating such sophisticated data is a difficult task for human and are prone to error (Nasim et al. 2018). A study by Madahali and Hall (2020) shows that both bots and human in *gilani-17* have similar and unexpected behavior. Additionally, it is to be noted that, even the works that consider content-based and network-based features also perform comparatively lower on these two datasets (Sayyadiharikandeh et al. 2020). The performance of a supervised machine learning model largely depends on the data it is trained on. However, we can say that, by using the deep learning approach, we are able to improve the performance and generalizability of the model even by using a reduced set of features.

#### Performance analysis of the model

**Fig. 10:**
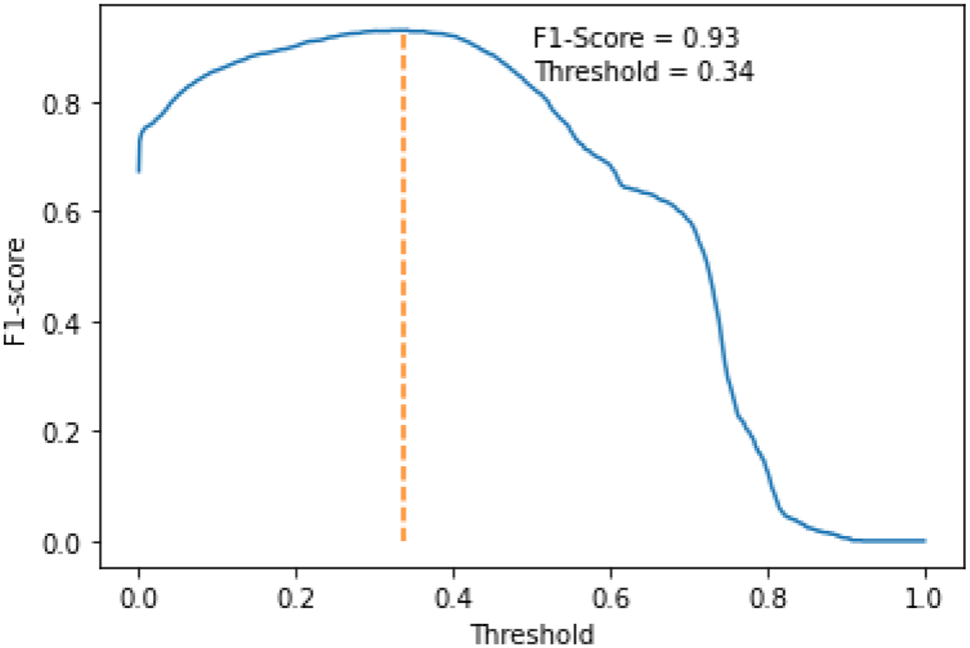
F1-scores for different threshold values

The model achieves an accuracy of 0.92 and an F1-score of 0.83 on the hold-out dataset. A fine-tuned selection of threshold maximizes the F1-score. F1-scores for different threshold values are given in Fig. 10. The threshold of 0.34 gives an F1-score of 0.93. The results open a scope for improvement in its accuracy value.

**Fig. 11:**
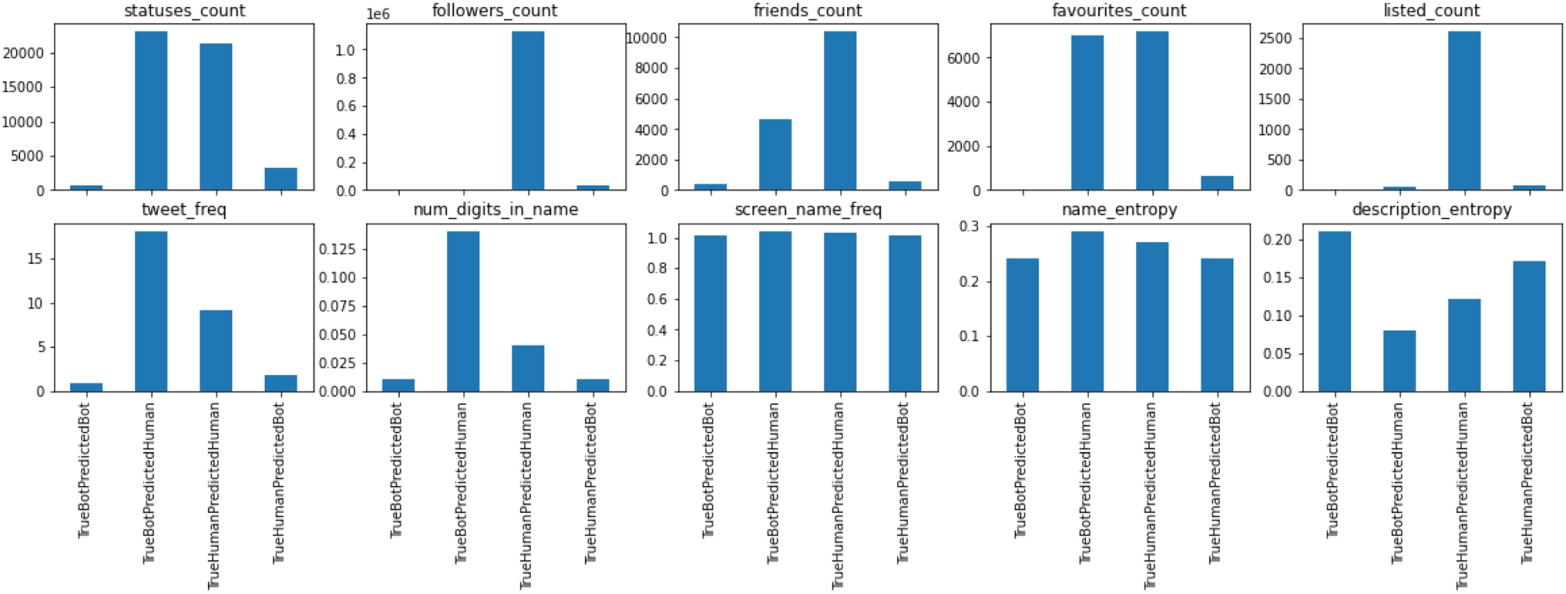
Average feature values across groups of correctly classified and misclassified test data

A further analysis on the classified data is shown in Fig. 11. Based on the predictions, we divided the whole test data into four groups. (a) Group of bot accounts that are predicted correctly as bots (TrueBotPredictedBot), (b) Group of bots that are predicted as human (TrueBotPredictedHuman), (c) Group of human accounts predicted correctly as human (TrueHumanPredictedHuman) and (d) Group of human accounts predicted as bot (TrueHumanPredictedBot). Here, groups ‘b’ and ‘d’ represent the misclassified data. It can be seen that a larger *statuses_count* or *favourites_count* favor the prediction of human. As a result, bots with higher counts for these features are misclassified. Looking back to our training data, it lacks a good representation of bots with higher counts for these features. In training data, the average *statuses_count* for human is 18145, whereas that of bot is 2784. A better representation of all types of bots in the training dataset can further improve the accuracy of the model. We also did an example-based analysis on the description feature of the classified data. Examples of description feature from correctly classified data include:

Human: ‘*A senior accounting taxation professional inspired chief accountant.*

Bot: ‘*Internet marketing help succeed making money online.’*

Considering the above examples, the model could distinguish an individual-centered description from that of a promotional description. Analyzing a misclassified sample, where a bot is classified as human, has the following description:

‘I’m poor bot I need sympathy.’

Here, even though description text is individual-centered, the semantics of the description clearly states that it is a bot. However, the model failed to capture it and wrongly classified the sample as human. This leaves the scope for a better embedding of description text for a better classification. Another potential limitation of the model is regarding its usability in multilingual settings. However currently our work focuses on analyzing the significance of including description as a feature to detect bots. Using a multilingual framework for text feature processing can take the work to the next level.

## Conclusion

This paper has proposed a novel framework, DeeProBot, which uses deep learning technique to detect bots from the user profile metadata-based features from Twitter. Using only the profile-based features including the description text to detect bots is a novel idea put forward in this work. The proposed framework consists of preparing the training and test datasets, feature engineering and the deep neural network design for detecting bots. Using the description text improved the cross-domain performance of the model across the test datasets. We have used the backward elimination feature selection in selecting the best subset of features that make the model to perform better with a lower-dimensional feature set. Also, embedding the text feature using GLoVe helped in better learning from the feature. DeeProBot uses a hybrid DNN model to detect bots, where the description feature was learned using the LSTM units and the rest of the features were learned using the dense layers. The generalizability of the model is preserved by adding activity regularizer to LSTM layers and by adding dropout layers in between the dense layers. Further, early stopping helps in stopping the training process when the model starts overfitting. We evaluated the performance of the model against a 20% hold-out subset of the same dataset used for training and we evaluated the cross-domain performance of the model by testing the model on four separate test datasets. DeeProBot could detect bots with an AUC of 0.97 on the hold-out test set. Also, the model obtained a better generalization than that of baseline with a reduced set of features.

One of our plans for future work involves testing the detection of bots with a real-time stream of Twitter data which also gives us an analysis on the scalability of DeeProBot. Also, bots with new behavioral patterns are evolving day by day, making it necessary to come up with a model that can capture these changing patterns. As a future work, we plan to design the model so as to learn these behavioral patterns. Furthermore, it remains to be seen whether with an extra overload of extracting other types of features like content-based and interaction-based features, the performance of the model can be improved.

## Footnotes

1 https://botometer.osome.iu.edu/bot-repository

2 https://nlp.stanford.edu/projects/glove
